# Differentiation between depressive disorder and insomnia disorder with depressive symptoms across age groups using machine learning

**DOI:** 10.3389/fpsyt.2026.1783381

**Published:** 2026-08-20

**Authors:** Xiaona Zhang, Ning Ren, Mingjian Cai, Mingfen Song, Hongjing Mao

**Affiliations:** Affiliated Mental Health Center, Zhejiang University School of Medicine(Hangzhou Seventh People’s Hospital), Hangzhou, China

**Keywords:** age-related symptom, depressive disorder, feature importance analysis, insomnia disorder with depressive symptoms, machine learning

## Abstract

**Objective:**

To investigate symptom features distinguishing depressive disorder from insomnia disorder with depressive symptoms across different age groups, and to provide evidence for refined clinical differential diagnosis.

**Methods:**

This retrospective cross-sectional study enrolled patients who visited Hangzhou Seventh People’s Hospital between January 2016 and October 2024 and completed assessments via the “Good Sleep 365” platform. Based on DSM-5 diagnostic criteria, two psychiatrists classified participants into two diagnostic groups: depressive disorder and insomnia disorder with depressive symptoms. The final sample included 1,873 patients: 190 adolescents, 1,253 adults, and 430 older adults. After an 8:2 training-validation split, feature selection and model training were performed within the training set, and final performance was evaluated on the validation set. Age-specific logistic regression models were constructed using Pittsburgh Sleep Quality Index (PSQI) component scores and item-level data from the 9-Item Patient Health Questionnaire (PHQ-9) and 15-Item Patient Health Questionnaire (PHQ-15), with feature importance analyses performed.

**Results:**

The two groups differed in demographic characteristics and total scale scores, although their overall clinical presentations showed substantial overlap. Depressed mood, fatigue, impaired concentration, and sleep-related symptoms consistently emerged across age groups, demonstrating relatively stable discriminative value, while distinct age-specific symptom patterns were also observed. Model performance varied across age groups, with the highest AUC observed in adults (0.8216), followed by older adults (0.7446) and adolescents (0.6229).

**Conclusion:**

Depressive disorder and insomnia disorder with depressive symptoms exhibit identifiable differences in symptom structure that are age-related. Symptom-level evaluation across age groups may provide complementary information for clinical differential diagnosis between these two conditions.

## 1 Introduction

Depressive disorder is one of the leading mental disorders worldwide, contributing substantially to functional impairment and social burden, and consistently ranks among the top causes of global disability (1). Its clinical manifestations encompass multiple dimensions, including cognitive, affective, somatic, and social functioning, thereby significantly impairing quality of life and psychosocial functioning (2). Meanwhile, insomnia disorder is also highly prevalent globally, with epidemiological studies reporting an overall prevalence of approximately 16.2%, and a prevalence of severe insomnia reaching up to 7.9% (3). Insomnia is highly comorbid with various mental disorders, particularly depressive and anxiety disorders (4), and is commonly accompanied by symptoms such as fatigue, impaired attention, and emotional instability (5).

Insomnia is widely regarded as one of the most common and clinically significant symptoms of depressive disorder and plays an important role in its onset, course, and prognosis (6). A growing body of evidence suggests that insomnia is not only a core symptom of depressive disorder but may also function as a prodromal factor and a predictor of its development (7–9). Individuals with insomnia have a significantly increased risk of developing depressive disorder (10, 11), whereas effective interventions targeting insomnia have been shown to reduce the incidence of major depressive disorder (12).

However, in clinical practice, insomnia disorder and depressive disorder show substantial overlap at the symptom level. Some patients with insomnia disorder may present with elevated scores on depression rating scales such as the 9-Item Patient Health Questionnaire (PHQ-9); however, after a comprehensive psychiatric evaluation, they do not meet the diagnostic criteria for depressive disorder and are instead diagnosed with insomnia disorder. Previous research has shown substantial overlap in depressive symptoms between patients with insomnia and those with major depressive disorder, including insomnia, fatigue, and attention problems (13). These findings suggest that total depression scale scores alone may be insufficient for differential diagnosis in patients with prominent insomnia symptoms, and that symptom-level analysis may provide more clinically useful information.

Age may further influence the clinical presentation of depressive and insomnia-related symptoms. Previous studies have shown that adolescents, adults, and older adults may differ in depressive symptom profiles, including vegetative, cognitive-affective, psychomotor, and somatic symptoms (14, 15). However, few studies have examined whether symptom-level features distinguishing depressive disorder from insomnia disorder with depressive symptoms vary across age groups. Therefore, it is necessary to conduct age stratification and symptom item-level analysis. In addition to age, demographic and clinical factors such as sex and education level may also be associated with insomnia and depressive symptom presentation (16).

In recent years, machine learning approaches have been increasingly applied to the screening, prediction, and auxiliary diagnosis of mental disorders due to their advantages in handling multidimensional and high-dimensional data (17, 18). However, systematic investigations focusing on the distinguishing features between insomnia disorder and depressive disorder across different age stages remain relatively limited.

Based on this background, the present study utilized real-world clinical data and applied machine learning algorithms to distinguish depressive disorder from insomnia disorder with depressive symptoms at the symptom-item level, using data derived from the PHQ-9, Pittsburgh Sleep Quality Index (PSQI), and 15-Item Patient Health Questionnaire (PHQ-15). By conducting age-stratified modeling and feature importance analyses in adolescent, adult, and older adult populations, this study further aimed to identify core symptom features with stable or age-specific discriminative value across different age stages. Age stratification was used to examine differences in symptom patterns across clinically meaningful age groups. This approach may provide evidence for clinical identification, refined assessment, and individualized intervention.

A related Chinese-language article based on the same research project has previously been published by our group (19). The previous study developed and evaluated a machine-learning model to differentiate depressive disorder from insomnia disorder with depressive symptoms in patients aged 18–65 years. The present study extends this work by investigating age-related differences in discriminative symptom profiles using age-stratified machine-learning models. Although the two studies share partially overlapping study populations, they address different research questions and report distinct findings.

## 2 Research methods and design

### 2.1 Study subjects

This study was a retrospective cross-sectional study. Patients who attended the “Sleep Disorder” outpatient clinic of Hangzhou Seventh People’s Hospital between January 1, 2016, and October 9, 2024, were included. Patients could attend the clinic either through self-referral or referral from other clinical departments. All participants completed a general information questionnaire, PSQI, PHQ-9, and PHQ-15 through the “Good Sleep 365” platform. The PSQI (20) is a widely used self-report instrument for assessing sleep quality and sleep disturbances over the previous month. It contains 19 self-rated items that are grouped into seven component scores. The total score ranges from 0 to 21, with higher scores indicating poorer sleep quality. The PHQ-9 (21) is a nine-item self-report questionnaire used to assess depressive symptom severity over the previous two weeks. The total score ranges from 0 to 27, with higher scores indicating more severe depressive symptoms. The PHQ-15 (22) is a 15-item self-report questionnaire designed to assess the severity of common somatic symptoms. The total score ranges from 0 to 30, with higher scores indicating greater somatic symptom burden. In this study, the PSQI was used as a general measure of sleep quality and sleep-related disturbances, rather than as a diagnostic instrument for insomnia disorder.

Diagnoses were made by two experienced psychiatrists according to the Diagnostic and Statistical Manual of Mental Disorders, Fifth Edition (DSM-5). The diagnostic process was based on routine clinical psychiatric assessment, clinical interviews, medical history, and available scale information. Patients were divided into two groups: the depressive disorder group and the insomnia disorder with depressive symptoms group, defined as meeting the DSM-5 diagnostic criteria for “insomnia disorder” and having a PHQ-9 score ≥ 10. Patients were further stratified into three age groups: adolescents (≤18 years), adults (19–54 years), and older adults (≥55 years) (23).

Initially, 1,243 patients with depressive disorder and 1,004 patients with insomnia disorder with depressive symptoms were identified. Patients with missing or incomplete questionnaire data were excluded, and a complete-case analysis was performed. Imputation was not conducted because this retrospective analysis was based on complete-case modeling, and complete item-level questionnaire data were required for feature selection and model construction. The final sample included 1,873 patients, including 991 patients with depressive disorder and 882 patients with insomnia disorder with depressive symptoms. By age group, the numbers of patients with depressive disorder and insomnia disorder with depressive symptoms were 135 and 55 in adolescents, 635 and 618 in adults, and 221 and 209 in older adults, respectively. The corresponding male/female distributions were 40/95 and 18/37 in adolescents, 182/453 and 153/465 in adults, and 75/146 and 54/155 in older adults. The study population in the present study partially overlaps with that of our previously published Chinese-language article (19). Compared with the previous study, the present study includes a broader age range and focuses on different research objectives and analytical strategies.

### 2.2 Inclusion and exclusion criteria

Inclusion criteria: 1. Meeting the DSM-5 diagnostic criteria for depressive disorder or insomnia disorder; 2. Having sufficient comprehension to complete the self-report questionnaires reliably, defined in this study as having at least a primary school education level and being able to complete the PSQI, PHQ-9, and PHQ-15 assessments in full; 3. In the insomnia disorder group, a PHQ-9 score ≥ 10.

Exclusion criteria: 1. Presence of severe physical illness; 2. History of alcohol or substance abuse; 3. Pregnant or breastfeeding women; 4. Other primary psychiatric diagnoses include bipolar disorder, schizophrenia, substance-related disorder, or anxiety disorder. Anxiety symptoms alone were not used as an exclusion criterion.

### 2.3 Statistical analysis and machine learning modeling

#### 2.3.1 Data preprocessing

Before analysis, all raw scale data underwent necessary cleaning and coding. Patients with missing values in any of the scales were excluded. The dataset was randomly split into a training set and a validation set in an 8:2 ratio before feature selection and model construction. Feature selection and model training were performed only within the training set. The validation set was used only for final model performance evaluation.

#### 2.3.2 Data analysis

Demographic characteristics were compared between the two diagnostic groups within each age stratum. Total scores were compared between the two diagnostic groups in the overall sample. Continuous variables were expressed as means and standard deviations, and categorical variables as numbers and percentages. Independent sample t-tests were used to compare differences between groups for continuous variables, while chi-square tests were applied for categorical variables. Analyses were conducted using IBM SPSS Statistics (version 27.0). All reported p-values were two-tailed, with statistical significance set at 0.05.

### 2.3.3 Model construction and training

The input variables included 30 symptom-level or component-level variables in total: seven PSQI component scores, nine PHQ-9 items, and fourteen PHQ-15 items. Item 4 of the PHQ-15 (“menstrual problems”) was not included in the main mixed-sex models because this sex-specific item was not applicable to male participants, and no unified coding approach was used in the present analysis. Logistic regression models were employed to classify depressive disorder and insomnia disorder with depressive symptoms. Logistic regression models were fitted with L2 regularization, with the inverse regularization strength parameter set to C = 0.1, to reduce overfitting and improve model stability. Before model fitting, all numerical predictors were standardized using z-score normalization to ensure that variables measured on different scales were comparable in the L2-regularized logistic regression models. To examine age-related differences in symptom discriminative patterns, separate models were independently constructed for the adolescent, adult, and older adult groups, thereby minimizing the influence of age-related heterogeneity in symptom distributions.

### 2.4 Feature importance analysis

To identify core symptom features that contributed most to differentiating the two conditions across different age stages, feature importance rankings were derived based on the magnitude of coefficients from the logistic regression models. On this basis, the top ten symptom items in each age group were selected as representative discriminative features for subsequent analyses.

To assess potential multicollinearity among the predictors included in the final models, variance inflation factors (VIF) were calculated separately for each age-specific logistic regression model. VIF analysis was performed using the ten symptom-level predictors retained in the final model for each age group. A VIF value below 5 was considered to indicate the absence of severe multicollinearity among predictors.

#### 2.4.1 Model performance evaluation

Model performance was evaluated on the validation set. Comprehensive classification metrics, including AUC, accuracy, precision, recall, and F1-score, were used for assessment:

Area under the receiver operating characteristic curve (AUC): A global indicator of the model’s ability to discriminate between positive and negative samples; values closer to 1 indicate better performance.

Accuracy: The proportion of correctly classified samples among all samples.

Precision: The proportion of true positives among samples predicted as positive, reflecting the accuracy of positive identification.

Recall (Sensitivity): The proportion of true positives correctly identified among all actual positive samples, reflecting the model’s ability to detect all positives.

F1-score: The harmonic mean of precision and recall, balancing both measures, especially suitable for imbalanced datasets.

These indicators collectively provided a comprehensive understanding of the model’s discriminative capacity across different age groups.

#### 2.4.2 Ethical approval

Due to the retrospective nature of the study, the Ethics Committee of the Institutional Review Board of Hangzhou Seventh People’s Hospital (ethical approval number:2025-005) waived the need for obtaining informed consent. The experimental protocol was approved by the Ethics Committee of the Institutional Review Board of Hangzhou Seventh People’s Hospital, and all methods were carried out in accordance with relevant guidelines and regulations.

## 3 Results

### 3.1 Demographic characteristics

Among adolescents, no significant differences were found between the two groups in terms of age (P = 0.528), gender (P = 0.674), education level (P = 0.866), or duration of insomnia (P = 0.596) (see **Table 1**).

**Table 1:** Demographic characteristics of adolescents.

Characteristic	Depressive disorder group, N (%)	Insomnia disorder group, N (%)	χ²/t	P
Age	16.18 ± 1.32	16.31 ± 1.25	-6.632	0.528
Gender			0.177	0.674
Male	40 (29.6%)	18 (32.7%)		
Female	95(70.4%)	37(67.3%)		
Education level			0.029	0.866
Primary school	0(0%)	0(0%)		
Secondary school	133(98.5%)	54(98.2%)		
College and above	2(1.5%)	1(1.8%)		
Duration of insomnia			3.681	0.596
<1month	35(25.9%)	13 (23.6%)		
1-3 months	31(23.0%)	12 (21.8%)		
3 months-1year	46(34.1%)	16 (29.1%)		
1-3years	16(11.9%)	10 (18.2%)		
3-5years	6(4.4%)	2 (3.6%)		
5-10years	1(0.7%)	2(3.6%)		

t denotes the independent-samples t-test statistic; χ² denotes the chi-square test. P < 0.05 was considered statistically significant.

Among adults, no significant difference was observed between the two groups in terms of gender (P = 0.118). However, significant differences were found in age (P < 0.001), education level (P < 0.001), and duration of insomnia (P < 0.001). Specifically, the average age of the insomnia disorder group was higher than that of the depressive disorder group. A higher proportion of patients in the depressive disorder group had secondary school or college and above education, while the insomnia disorder group had a higher proportion of patients with only primary school education, indicating that the overall educational attainment of the depressive disorder group was higher. In terms of insomnia duration, the proportion of patients with insomnia lasting less than one year was higher in the depressive disorder group, whereas the insomnia disorder group had a larger proportion of patients with insomnia lasting more than one year, suggesting that insomnia tended to persist longer in this group (see **Table 2**).

**Table 2:** Demographic characteristics of adults.

Characteristic	Depressive disorder group, N (%)	Insomnia disorder group, N (%)	χ²/t	P
Age	34.27 ± 10.49	39.37 ± 9.62	-8.966	<0.001*
Gender			2.437	0.118
Male	182 (28.7%)	153 (24.8%)		
Female	453(71.3%)	465(75.2%)		
Education level			20.259	<0.001*
Primary school	38(6.0%)	65(10.5%)		
Secondary school	180(28.3%)	220(35.6%)		
College and above	417(65.7%)	333(53.9%)		
Duration of insomnia			78.183	<0.001*
<1 month	101(15.9%)	54 (8.7%)		
1-3 months	135(21.3%)	83 (13.4%)		
3 months-1year	165(26.0%)	114 (18.4%)		
1-3 years	88(13.9%)	112 (18.1%)		
3-5 years	66(10.4%)	74 (12.0%)		
5-10 years	80(12.6%)	181(29.3%)		

t denotes the independent-samples t-test statistic; χ² denotes the chi-square test. *P < 0.05 was considered statistically significant.

Among older adults, no significant differences were found between the two groups in terms of gender (P = 0.067) or education level (P = 0.566). However, significant differences were observed in age (P < 0.001) and duration of insomnia (P < 0.001). Specifically, the average age of the depressive disorder group was higher than that of the insomnia disorder group. A higher proportion of patients in the depressive disorder group had secondary school or college and above education, while the insomnia disorder group had a higher proportion of patients with only primary school education, indicating that the overall educational attainment of the depressive disorder group was higher. Regarding insomnia duration, the proportion of patients with insomnia lasting less than three years was higher in the depressive disorder group, whereas the insomnia disorder group had a larger proportion of patients with insomnia lasting more than five years, suggesting that insomnia tended to persist longer in this group (see **Table 3**).

**Table 3:** Demographic characteristics of older adults.

Characteristic	Depressive disorder group, N (%)	Insomnia disorder group, N (%)	χ²/t	P
Age	62.98 ± 6.20	60.92 ± 5.44	3.658	<0.001*
Gender			3.355	0.067
Male	75 (33.9%)	54 (25.8%)		
Female	146(66.1%)	155(74.2%)		
Education level			1.139	0.566
Primary school	73(33.0%)	79(37.8%)		
Secondary school	108(48.9%)	93 (44.5%)		
College and above	40(18.1%)	37(17.7%)		
Duration of insomnia			27.957	<0.001*
<1month	24(10.9%)	16 (7.7%)		
1-3months	41(18.6%)	31 (14.8%)		
3months-1 year	59(26.7%)	27 (12.9%)		
1-3 years	34(15.4%)	31 (14.8%)		
3-5 years	20(9.0%)	20 (9.6%)		
5-10 years	43(19.5%)	84(40.2%)		

t denotes the independent-samples t-test statistic; χ² denotes the chi-square test. *P < 0.05 was considered statistically significant.

Demographic analyses indicated that, in the adolescent group, no significant differences were observed between the two patient groups with respect to age, sex, educational level, or duration of insomnia, suggesting a high degree of demographic homogeneity between the two conditions at this stage. In contrast, among adult and older adult patients, the insomnia disorder group exhibited a longer duration of insomnia, whereas patients with depressive disorder had relatively higher educational attainment, indicating that with increasing age, the two conditions show a gradual divergence in sociodemographic characteristics and illness course.

### 3.2 Comparison of total scale scores

The PSQI scores of the two groups were 13.32 ± 3.42 and 13.95 ± 2.61, respectively, showing a significant difference (P < 0.001). This indicates that the insomnia disorder group had higher PSQI scores than the depressive disorder group, suggesting poorer sleep quality. The PHQ-9 scores of the two groups were 19.05 ± 4.44 and 13.80 ± 3.79, respectively, also showing a significant difference (P < 0.001). This indicates that the depressive disorder group had higher PHQ-9 scores than the insomnia disorder group, reflecting more severe depressive symptoms. The PHQ-15 scores of the two groups were 11.64 ± 4.23 and 10.10 ± 3.78, respectively, with a significant difference (P < 0.001). This indicates that the depressive disorder group had higher PHQ-15 scores than the insomnia disorder group, suggesting a heavier somatic symptom burden (see **Table 4**).

**Table 4:** Baseline total scores of the two groups.

Scale scores	Depressive disorder group, mean ± SD	Insomnia disorder group, mean ± SD	t	P
PSQI	13.32 ± 3.42	13.95 ± 2.61	27.405	<0.001*
PHQ-9	19.05 ± 4.44	13.80 ± 3.79	-4.461	<0.001*
PHQ-15	11.64± 4.23	10.10± 3.78	-4.530	<0.001*

Total Scores of the Two Groups [Mean ± Standard Deviation (SD)]. t denotes the independent-samples t-test statistic; *P < 0.05 was considered statistically significant.

### 3.3 Results of feature importance analysis

**Figure 1:**
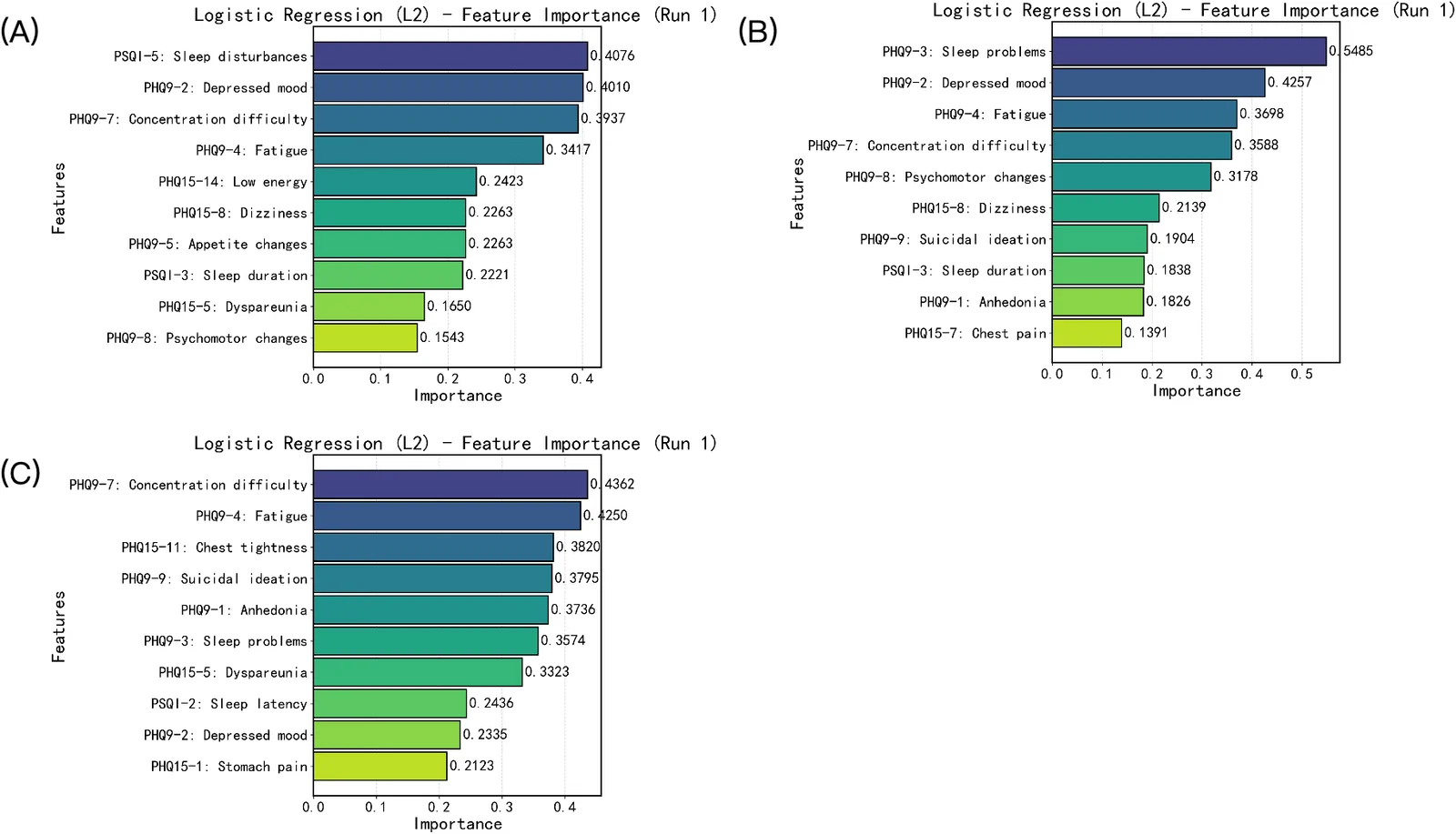
Results of feature importance analysis across different age groups: **(A)** adolescents, **(B)** adults, **(C)** older adults.

Based on the feature importance ranking from the logistic regression model, the top 10 items with the greatest ability to distinguish between the two patient groups were extracted for each age group (see **Figure 1**).

Feature importance analysis identified both shared and age-specific discriminative symptom patterns across the three age groups. Across age groups, depressed mood, fatigue or low energy, concentration difficulty, and sleep-related symptoms repeatedly appeared among the most important features, suggesting relatively stable discriminative value. In adolescents, the important features included both affective and cognitive symptoms, such as depressed mood and concentration difficulty, as well as sleep-related and somatic symptoms, such as sleep disturbances, sleep duration, dizziness, appetite changes, and dyspareunia. In adults, the important features were mainly typical depressive symptoms, including depressed mood, fatigue, concentration difficulty, psychomotor changes, suicidal ideation, and anhedonia, together with sleep problems and somatic symptoms such as dizziness and chest pain. In older adults, concentration difficulty, fatigue, suicidal ideation, anhedonia, and sleep-related symptoms remained important, while somatic symptoms such as chest tightness, dyspareunia, and stomach pain were more prominent. These findings suggest that symptom-level differences between depressive disorder and insomnia disorder with depressive symptoms vary by age group.

Multicollinearity diagnostics were further conducted for the predictors included in the final age-specific logistic regression models. All VIF values were below 5 across the three age groups, indicating that severe multicollinearity was not present among the retained predictors. The maximum VIF values were 1.594 in the adolescent group, 1.824 in the adult group, and 1.483 in the older adult group (see **Tables 5**–**7**).

**Table 5:** Multicollinearity diagnostics of adolescents.

Variable	Unstandardized coefficient		t	P	Multicollinearity diagnostics	
	B	SE			Tolerance	VIF
(Constant)	1.719	0.137	12.522	<0.001		
PSQI-5	-0.129	0.054	-2.397	0.018	0.759	1.317
PHQ9-2	-0.073	0.038	-1.934	0.055	0.627	1.594
PHQ9-7	-0.095	0.030	-3.162	0.002	0.844	1.185
PHQ9-4	-0.089	0.043	-2.085	0.039	0.708	1.412
PHQ15-14	0.107	0.046	2.314	0.022	0.920	1.088
PHQ15-8	0.131	0.057	2.284	0.024	0.718	1.393
PHQ9-5	-0.052	0.040	-1.300	0.195	0.705	1.418
PSQI-3	0.048	0.025	1.898	0.059	0.884	1.131
PHQ15-5	-0.056	0.042	-1.339	0.182	0.911	1.098
PHQ9-8	0.007	0.036	0.193	0.847	0.802	1.247

SE, standard error; VIF, variance inflation factor; PSQI, Pittsburgh Sleep Quality Index; PHQ9, 9-Item Patient Health Questionnaire; PHQ15, 15-Item Patient Health Questionnaire.

**Table 6:** Multicollinearity diagnostics of adults.

Variable	Unstandardized coefficient		t	P	Multicollinearity diagnostics	
	B	SE			Tolerance	VIF
(Constant)	2.100	0.074	28.462	<0.001		
PHQ9-3	-0.122	0.017	-7.310	<0.001	0.691	1.448
PHQ9-2	-0.063	0.015	-4.235	<0.001	0.693	1.443
PHQ9-4	-0.077	0.016	-4.673	<0.001	0.548	1.824
PHQ9-7	-0.064	0.011	-5.707	<0.001	0.862	1.160
PHQ9-8	-0.054	0.014	-3.910	<0.001	0.591	1.693
PHQ15-8	0.098	0.030	3.322	<0.001	0.910	1.099
PHQ9-9	-0.041	0.012	-3.331	<0.001	0.790	1.266
PSQI-3	0.038	0.011	3.532	<0.001	0.922	1.085
PHQ9-1	-0.032	0.012	-2.621	0.009	0.730	1.370
PHQ15-7	-0.047	0.027	-1.706	0.088	0.892	1.121

SE, standard error; VIF, variance inflation factor; PSQI, Pittsburgh Sleep Quality Index; PHQ9, 9-Item Patient Health Questionnaire; PHQ15, 15-Item Patient Health Questionnaire.

**Table 7:** Multicollinearity diagnostics of older adults.

Variable	Unstandardized coefficient		t	P	Multicollinearity diagnostics	
	B	SE			Tolerance	VIF
(Constant)	2.151	0.098	21.934	<0.001		
PHQ9-7	-0.061	0.018	-3.508	<0.001	0.892	1.122
PHQ9-4	-0.112	0.024	-4.593	<0.001	0.674	1.483
PHQ15-11	0.085	0.031	2.788	0.006	0.954	1.049
PHQ9-9	-0.047	0.019	-2.391	0.017	0.792	1.262
PHQ9-1	-0.063	0.021	-3.011	0.003	0.700	1.428
PHQ9-3	-0.120	0.026	-4.616	<0.001	0.774	1.292
PHQ15-5	-0.067	0.027	-2.500	0.013	0.908	1.101
PSQI-2	0.033	0.026	1.236	0.217	0.980	1.020
PHQ9-2	-0.017	0.023	-0.728	0.467	0.787	1.270
PHQ15-1	0.026	0.027	0.934	0.351	0.955	1.048

SE, standard error; VIF, variance inflation factor; PSQI, Pittsburgh Sleep Quality Index; PHQ9, 9-Item Patient Health Questionnaire; PHQ15, 15-Item Patient Health Questionnaire.

### 3.4 Model validation and evaluation

**Figure 2:**
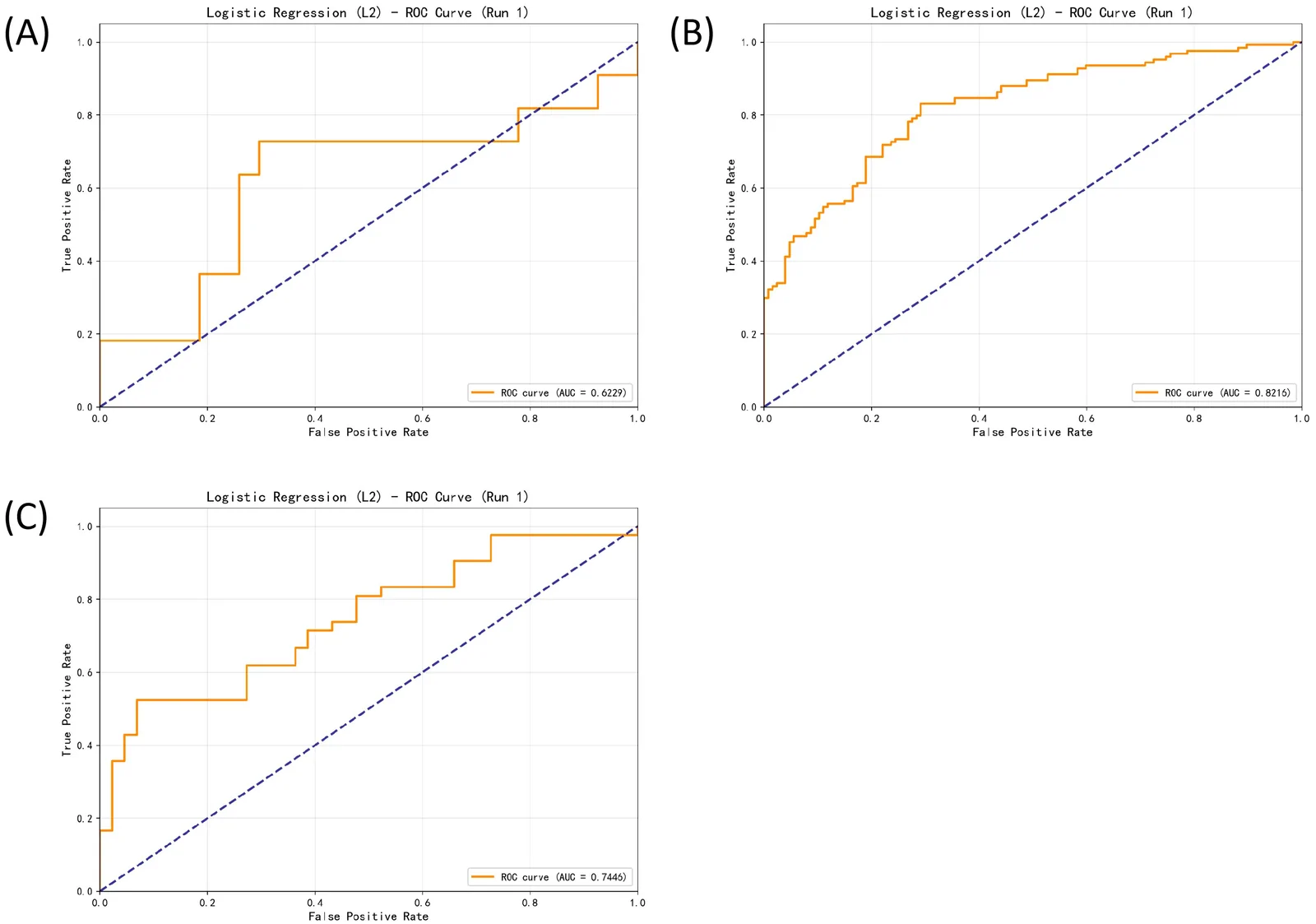
ROC curves across different age groups: **(A)** adolescents, **(B)** adults, **(C)** older adults.

**Figure 3:**
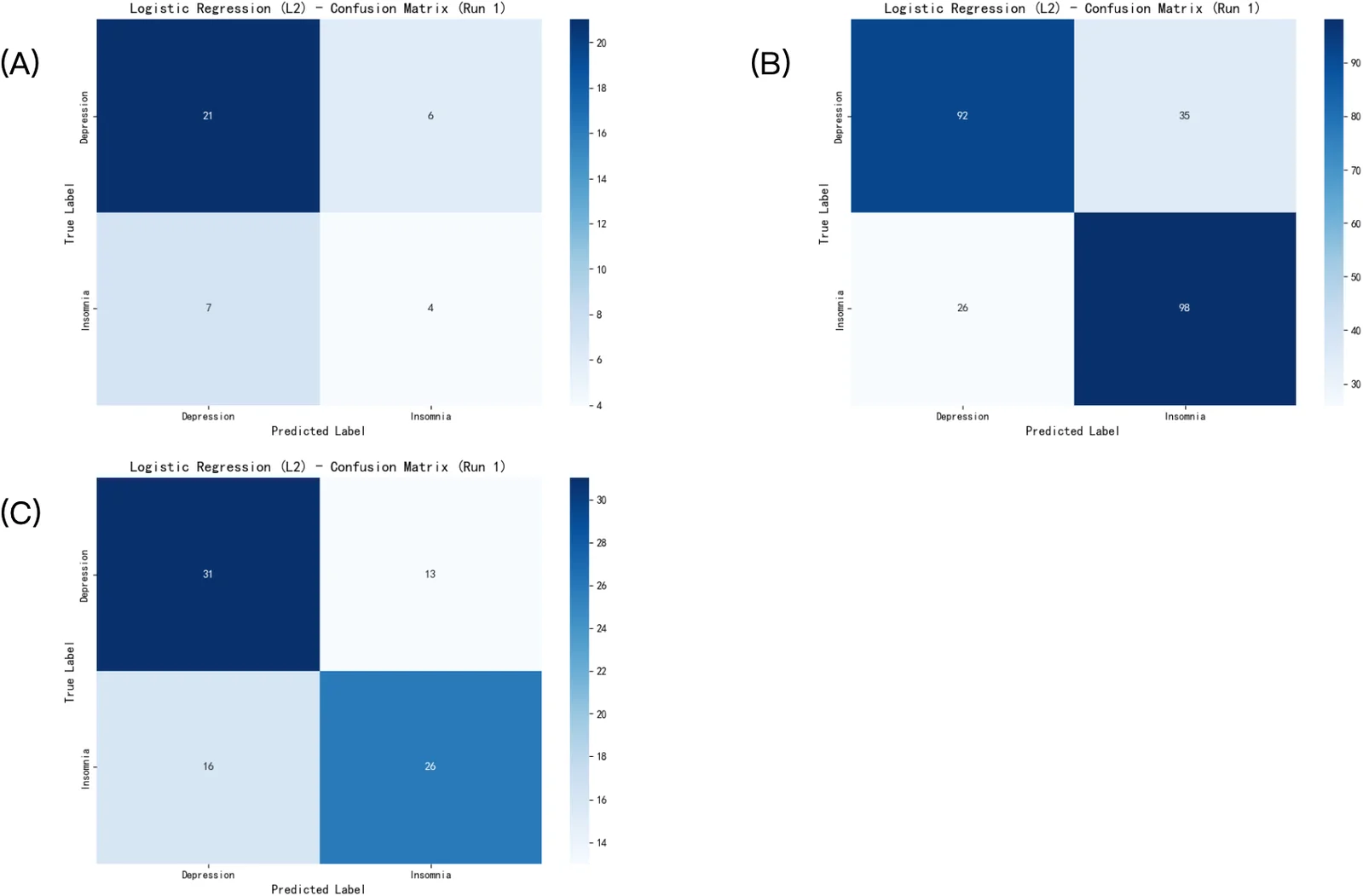
Confusion matrices across different age groups: **(A)** adolescents, **(B)** adults, **(C)** older adults.

The final models constructed using the selected symptom features were evaluated on the validation set (see **Figures 2**, **3**). All performance metrics, including AUC, accuracy, precision, recall, and F1-score, are reported in **Table 8**. The adult model showed the highest AUC (0.8216), followed by the older adult model (0.7446) and the adolescent model (0.6229). These results indicate that model performance varied across age groups, although the influence of unequal sample sizes should be considered.

**Table 8:** Model performance across different age groups.

Age	AUC	Accuracy	Precision	Recall	F1-score
Adolescents	0.6229	0.6579	0.7500	0.7778	0.7636
Adult	0.8216	0.7570	0.7797	0.7244	0.7510
Older adults	0.7446	0.6628	0.6596	0.7045	0.6813

### 3.5 Logistic regression model parameters

The following equation is provided to describe how predicted probabilities were calculated in the fitted logistic regression models. If these models are applied to other observations in future research, the same predictors should be extracted and coded in the same manner as in the training data.


$$\text{z}\;=\;\text{intercept}\;+\;\text{Σ}\;\left(\text{coefficien}{\text{t}}_{\text{i}}\;\times \;\text{featur}{\text{e}}_{\text{i}}\right)$$

The linear predictor was subsequently transformed into a predicted probability using the sigmoid function:


$$\text{P}\;=\;1/(1\;+\;{\text{e}}^{{-}_{\text{z}}})$$

A probability threshold of P ≥ 0.5 was applied to classify samples as depressive disorder; otherwise, samples were classified as insomnia disorder with depressive symptoms. The intercept values for the models were -0.812551 for adolescents, -0.080599 for adults, and -0.149273 for older adults.

## 4 Discussion

This study, based on real-world clinical assessment data from the “Good Sleep 365” platform, systematically explored symptom expression patterns distinguishing depressive disorder from insomnia disorder with depressive symptoms across different age groups by integrating scale-based assessments and machine learning modeling, and constructed classification models to differentiate the two conditions. The results demonstrated that although the two disorders show substantial overlap in overall clinical presentation, clinically meaningful differences remain in symptom structure, scale scores, and demographic characteristics, with these differences exhibiting clear age-related patterns. Specifically, both relatively stable core discriminative symptoms across age groups and distinct age-specific symptom patterns were identified. Although the PSQI score was significantly higher in the insomnia disorder with depressive symptoms group, the absolute difference was small and should be interpreted cautiously in terms of clinical relevance.

Previous machine-learning studies have examined the relationship between sleep problems and depression, mainly by predicting depression risk or depressive symptom severity from sleep-related variables (24, 25). These studies highlight the value of machine-learning approaches for understanding the close relationship between sleep disturbance and depression. In contrast, the present study focused on differentiating depressive disorder from insomnia disorder with depressive symptoms at the symptom-item level and further examined whether discriminative symptom patterns differed across age groups. This approach may provide complementary information for clinical differential diagnosis.

The performance of the models varied across age groups, with the highest AUC observed in adults. This may suggest that symptom-level differences are more distinguishable during adulthood. However, this interpretation should be made cautiously, because the adult group also had the largest sample size, which may have contributed to greater model stability and better estimated performance. In contrast, the relatively lower performance in adolescents may be partly attributable to the smaller sample size, which could have limited model stability, as well as to the more atypical or internalizing presentation of emotional and cognitive symptoms during this developmental period (26). In addition, as the present study was based on cross-sectional data, diagnostic trajectories could not be assessed. Some adolescent patients may experience diagnostic evolution during follow-up, such as progression to bipolar disorder or other affective disorders, which may partly affect the discriminative performance of models derived from cross-sectional data. The model performance in the older adult group was moderate, suggesting that traditional affective symptom dimensions may have limited discriminative utility in older adults (27).

These findings suggest that symptom-level features may provide useful information for differentiating the two conditions across age groups. However, the age-related differences in model performance, especially in adolescents, should be interpreted cautiously because the smaller sample size and diagnostic-group imbalance may have limited model stability and feature robustness. Therefore, age-specific symptom patterns should be considered as preliminary and require further validation in larger and more balanced samples.

Insomnia is not only an important symptom of depressive disorder but may also function as a neurobiological prodromal mechanism. The hyperarousal model suggests that insomnia is associated with cortical hyperactivation, elevated nocturnal cortisol levels, and increased sympathetic nervous system activity, which may subsequently disrupt the functioning of key emotion-regulation brain regions, such as the prefrontal cortex and the hippocampus (28). Interventions targeting insomnia may, to some extent, alleviate depressive symptoms (29), and may also improve response to antidepressant treatment (30). Previous evidence from neurobiological models and intervention studies suggests that insomnia may be more than a symptom of depressive disorder and could represent a clinically relevant therapeutic target. It should also be noted that sleep disturbances in depressive disorder are heterogeneous. Although insomnia is common in depression, hypersomnolence-related symptoms, including hypersomnia and excessive daytime sleepiness, may also be clinically relevant in patients with major depressive disorder (31).

Previous studies have indicated that cognitive impairment may persist long after the remission of affective symptoms (32). Declines in cognitive function have been shown to be associated with both fatigue and sleep disturbances (33). Fatigue, depression, and cognitive impairment frequently co-occur, with a particularly close relationship between depression and fatigue, and their effects on cognitive functioning appear to substantially overlap (34). These symptoms may also share common neurobiological mechanisms, including inflammation-related pathways (35, 36). In the present study, impaired concentration and fatigue repeatedly emerged among the most important discriminative features across multiple age groups, further supporting these findings.

At the same time, age-specific symptom patterns were also evident. Among adolescents, appetite changes, dizziness, and sleep-related problems emerged as prominent discriminative features, which may reflect the atypical expression of emotional symptoms and a greater tendency toward somatization during this developmental stage. Appetite loss or overeating constituted particularly salient distinguishing features, with more than half of adolescents experiencing decreased appetite or engaging in “emotional eating” behaviors (14, 37, 38). In addition, among typical depressive symptoms, reduced interest or anhedonia was not prominent in the adolescent group. Previous studies have shown that core symptoms of interest loss or anhedonia are less frequently observed in adolescents than in adults (14).

Among adults, depressive disorder more commonly manifests with typical core depressive symptoms, such as depressed mood, loss of interest, and psychomotor changes, which facilitates differentiation from insomnia disorder with depressive symptoms at the level of symptom structure. In contrast, among older adults, somatic symptoms—including chest tightness, gastric pain, and pain during sexual activity—were particularly prominent. This pattern may reflect a tendency for older adults to express psychological distress through somatic complaints rather than subjective emotional experiences (27, 39), and older adults are known to experience a higher burden of pain symptoms compared with other age groups (40). Previous research has reported that approximately 75% of elderly women with depressive disorder experience sexual dysfunction (41), a domain that is frequently overlooked in clinical practice, highlighting the need for greater attention to sexual health in this population. Accordingly, clinical assessment in older adult patients should incorporate multiple dimensions beyond affective symptoms alone. Overall, the observed age-related differences in symptom expression suggest that clinical evaluation should prioritize different symptom domains according to age, rather than relying on a uniform assessment framework across the lifespan.

## 5 Conclusion

Based on real-world clinical data, this study combined item–level analysis with machine learning approaches to investigate age-related symptom differences between depressive disorder and insomnia disorder with depressive symptoms. The results indicate that although the two conditions show substantial overlap in overall clinical presentation, clinically meaningful differences remain at the level of symptom structure, exhibiting clear age-related patterns. Specifically, depressed mood, fatigue, impaired concentration, and sleep-related symptoms demonstrated relatively stable discriminative value across age groups, while distinct age-specific symptom patterns were also observed. These findings suggest that symptom-level evaluation across age groups may provide complementary information for differentiating depressive disorder from insomnia disorder with depressive symptoms.

## 6 Limitations

This study has several limitations. First, its retrospective cross-sectional design precludes causal inference and assessment of treatment-related changes; longitudinal studies are needed to examine symptom trajectories, diagnostic changes, and treatment responses. Second, self-reported questionnaires may introduce recall or response bias. Participants with missing or incomplete questionnaire data were excluded without imputation, which may have introduced selection bias, particularly because a relatively large proportion of patients with depressive disorder were excluded. We did not examine whether demographic or clinical characteristics differed between participants who were included in the final analytic sample and those who were excluded because of missing or incomplete data. Therefore, the extent to which the final sample differed from the broader initially eligible sample remains unclear, and the generalizability of the findings should be interpreted cautiously. Future studies should compare included and excluded participants and conduct imputation-based sensitivity analyses where appropriate. Medication use and objective sleep assessments, such as polysomnography, were not systematically available, so the effects of medications or other sleep disorders could not be fully excluded. Third, unequal sample sizes across age groups and diagnostic-group imbalance within some age strata, especially the small and imbalanced adolescent subgroup, may have affected model stability, performance estimates, and feature robustness. Fourth, PHQ-15 Item 4 (“menstrual problems”) was not included in the main models, which may have reduced the inclusiveness of somatic symptom assessment for female participants and omitted a clinically relevant symptom domain. Finally, the models were based on a single clinical population and used logistic regression for interpretability. Future studies should validate these findings in larger, multicenter, and more balanced samples, retain menstrual-related items using appropriate coding strategies or sex-specific analyses, and compare alternative modeling approaches. In addition, the robustness of feature importance should be further examined using model-agnostic methods, such as SHAP values or permutation-based feature importance, in independent datasets.

## Data Availability

The datasets generated and analyzed during the current study are not publicly available due to confidentiality and ethical restrictions but are available from the corresponding author on reasonable request.
